# The importance of sex dimorphism in liver metabolism and progressive liver diseases

**DOI:** 10.1186/s13293-025-00811-7

**Published:** 2025-12-27

**Authors:** Eva Kočar, Kaja Blagotinšek Cokan, Tinkara Kreft, Tadeja Režen, Damjana Rozman

**Affiliations:** 1 Centre for Functional Genomics and Bio-Chips, Institute of Biochemistry and Molecular Genetics, Faculty of Medicine, University of Ljubljana, Ljubljana, Slovenia; 2 Molecular Biology Laboratory, BIA Separations CRO, Labena Ltd., Ljubljana, Slovenia

**Keywords:** Liver, MASLD, MASH, Fibrosis, HCC, Sexual dimorphism, Animal models

## Abstract

The liver is a central metabolic organ with pronounced sex-specific differences shaped by sex hormones, sex chromosome-linked gene expression, ageing, and circadian rhythm. These factors influence disease susceptibility, progression, and treatment response, with notable differences between females and males in the prevalence, severity, and clinical outcomes of metabolic dysfunction-associated steatotic liver disease. This condition represents a growing global health burden that can progress to hepatocellular carcinoma, the second leading cause of cancer-related death worldwide. Despite this impact, sex remains an underexplored variable in liver research, and the molecular mechanisms by which sex influences disease development remain poorly understood. In this review, we examine the key determinants of sex differences in liver pathogenesis. We highlight the protective role of estrogen signaling in female liver metabolism, the increased vulnerability to disease progression after menopause, and the contribution of circadian regulation to sex-specific outcomes. We further discuss how the lack of systematic inclusion of both sexes in preclinical and clinical studies limits the identification of biomarkers and the development of effective therapeutic interventions. Incorporating sex as a biological variable is therefore essential to improve mechanistic understanding, translational relevance, and the personalization of treatment approaches. Particular emphasis is placed on animal models that reflect sex-specific liver physiology and pathophysiology, as these provide valuable frameworks for studying disease progression and testing targeted interventions. In summary, recognizing and integrating sexual dimorphism in liver metabolism is crucial to advancing prevention, diagnosis, and treatment strategies. Addressing sex differences is critical for developing accurate diagnostic tools and personalized therapeutic approaches, ultimately improving outcomes for both women and men with liver disease.

## Highlights

- The liver exhibits pronounced sexual dimorphism, influenced by sex hormones, sex chromosomes, ageing, and circadian regulation
- Estrogen signaling is protective for liver metabolism, while postmenopausal hormonal decline increases disease vulnerability
- Sex-dependent differences in lipid handling, xenobiotic metabolism, and immune responses shape the course of liver diseases
- Generating and weighing alternative hypotheses for apparent sex differences found in data can reveal the important role of gender-related social factors in shaping human biology
- Limited inclusion of both sexes in preclinical and clinical studies hampers biomarker discovery and therapeutic optimisation.
- Considering sex as a fundamental biological variable is essential for accurate disease modelling, diagnosis, and personalised treatment of liver disorders

## Background

Sexual diversity in human physiology and sex prevalence in disease represent a foundation of a large number of pathologies. Yet we are far from a full comprehension of the mechanisms underpinning these differences. According to current understanding, sex-specific metabolic physiology is shaped not only by hormonal influences but also by evolutionary pressures that established a close relationship between the liver and the reproductive system. As the major metabolic organ, the liver developed specialized functions, largely coordinated by estrogen signaling, to meet the demands of female reproduction. Throughout the reproductive cycle, hepatic estrogen receptors (ER) regulate lipoprotein and apolipoprotein synthesis to support follicle maturation, steroid production, and cholesterol recycling across the reproductive cycle [1]. Interestingly, hepatic ER requires both estrogen binding and nutrient-dependent stimulation for its full transcriptional activation, thus acting as a metabolic checkpoint that blocks reproductive progression when amino acid intake is insufficient [2]. During pregnancy, placental estrogens drive hepatic adaptations in lipoprotein profiles to supply cholesterol for placental steroid synthesis, mobilize fat and glucose to meet fetal energy demands, and upregulate of P450 enzymes to support waste elimination. Reflecting the interdependence between liver and ovary, the expression and activity of the ER are highest in these two organs, and ER transcriptional activity oscillates in synchrony across them throughout the reproductive cycle [1].

For over four decades, it has been recognized that hepatic metabolism differs markedly between males and females [3]. Microarray analyses in mouse models have reported that up to approximately 70% of all sex-biased genes identified across somatic tissues are expressed in the liver [4]. More recent RNA-seq studies have confirmed and expanded this finding, showing that the liver exhibits one of the strongest sex-differential transcriptomic signatures among non-reproductive organs. Bulk and single-cell RNA-seq analyses in mice reveal hundreds to thousands of sex-biased hepatic genes, highlighting the liver as the second most sexually dimorphic organ after the gonads [5]. Human RNA-seq datasets support these observations: large-scale GTEx-based transcriptomic analyses identify the liver as one of the most sexually dimorphic human somatic organs based on metabolic functions, with pronounced sex differences in xenobiotic metabolism, lipid homeostasis, and immune signaling [6, 7]. This underlies the well-recognized sex differences in drug metabolism, susceptibility to some liver pathologies, and lipid metabolism which were described already in animal models [8]. Further investigation into human hepatic sexual dimorphism is essential to unravel the molecular underpinnings of sex-specific disease etiology and to pave the way for personalized treatments that account for distinct metabolic and hormonal profiles in both, men and women.

In this review, we critically examine the molecular mechanisms driving hepatic sexual dimorphism in metabolic-associated fatty liver disease and detail how sex-specific differences in gene expression, hormonal signaling, and circadian regulation contribute to disease progression toward hepatocellular carcinoma (HCC). We also discuss emerging strategies for personalized therapeutic interventions.

## Sex-based metabolic dysfunction-associated steatotic liver disease (MASLD)

**Fig. 1:**
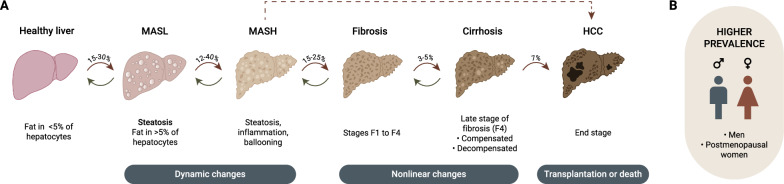
Progression of metabolic dysfunction-associated steatotic liver disease (MASLD) towards hepatocellular carcinoma (HCC) and its prevalence. **A** Liver steatosis is present in approximately 30% of the global adult population. In 12–40% of patients the latter progresses to MASH. Steatosis and MASH are characterized by bidirectional dynamic changes. When MASH persists (in 15–25% of the patients), it leads to the development of liver fibrosis. The stages of liver fibrosis, classified from F1 to F4, progress non-linearly. In some patients, fibrosis advances more rapidly, while in others, it progresses more slowly. Cirrhosis represents the late stage of fibrosis (F4) and is compensated. However, in 3–20% of the patients, it progresses to decompensated stage of cirrhosis. Approximately 7% of the patients with decompensated cirrhosis develop HCC. Red arrows indicate disease progression, while green arrows represent the potential for a reversible condition. **B** MASLD, MASH, fibrosis and HCC occur more frequently in men and in women after the menopause. *The figure was created in Adobe Illustrator. F, fibrosis stage; HCC, Hepatocellular Carcinoma; MASH, Metabolic Dysfunction-Associated Steatohepatitis; MASL, Metabolic Dysfunction-Associated Steatotic Liver*

Liver diseases caused by a large spectrum of metabolic manifestations are projected to rise in the forthcoming years [9–11] where systemic metabolic dysfunctions reflect in formation of Metabolic Dysfunction-Associated Steatotic Liver Disease (MASLD). This term replaces the acronym of Non-Alcoholic Fatty Liver Disease (NAFLD) that burdened the patients with stigma [10, 12, 13]. MASLD covers various clinical and pathological liver conditions differing in the degree of tissue damage and fibrosis and ultimately resulting in HCC [14–16]. While Metabolic Dysfunction-Associated Steatotic Liver (MASL) is considered as a reversible phenotype due to relatively benign accumulation of lipids in the hepatocytes (steatosis), Metabolic Dysfunction-Associated Steatohepatitis (MASH) is a more severe condition defined by a lobular inflammation and hepatocellular damage [17, 18] (Fig. 1).

MASLD is clinically highly heterogeneous in humans, indicating different patterns of liver steatosis, fibrosis, or cirrhosis [19]. In a recent study conducted on diverse genetic mouse models and in humans with MASLD and MASH [20], we identified a unique core transcriptional programme with inhibition of metabolic pathways and activated inflammatory pathways that associate with cancer. These findings exposed the importance of MASLD/MASH patient stratification to facilitate personalized liver disease management. Furthermore, for personalized understanding of MASLD complexity, a critical discussion about sex differences in MASLD is needed in basic research and clinical practice. There has been a resurgence of interest in artificial intelligence applications (AIs) supporting this sex-based challenge. AIs can integrate and synthesize a growing body of multidimensional data, deduce patterns, and predict outcomes to improve the investigation of prevalence, progression and prognosis of sex disparity in MASLD/MASLD-HCC [21–23].

Generally, sex should be considered as a biological variable in basic and clinical studies, as recently recommended by the Endocrine Society [24]. Sexual dimorphism is reflected by behavioral, physiological, and somatic differences between men and women, impacting the disease progression and outcome. The liver is the best example of sexually dimorphic non-reproductive organ in the body. It is not surprising that the prevalence, progression and severity of multiple liver diseases differ between men and women [16, 23, 25, 26]. Unfortunately, the impact of sex in liver research remains an under-characterized factor, as the understanding of molecular mechanisms by which sex modulates progression of MASLD in each sex is challenging [27, 28]. Benign liver lesions, alcohol-induced liver injuries and autoimmune liver diseases are more common in women. At the same time, men show higher susceptibility to malignant liver tumors, cholangitis, primary sclerosis and hepatitis infections. Prevalence of MASLD and MASH, considered as part of metabolic syndrome, is more likely in men and postmenopausal women [16, 26, 29–32]. MASH is the most common form of chronic liver disease since the groundbreaking discoveries of the hepatitis C virus (HCV), for which Harvey J. Alter, M. Houghton and C. M. Rice were awarded the Nobel Prize [33, 34], and represents a glimmer of hope in the ongoing fight against viral diseases.

Furthermore, MASH is the leading frontier for liver transplantation for women and the 2nd most common cause in men (following alcohol liver diseases) [35, 36]. Although liver transplantation has been the most effective treatment for HCC, the association between sex disparities and success of liver transplantation, in the context of the MASH-related HCC, remains a matter of intensive debate [37]. However, sex-based disparities in transplantation generally arise from a combination of physiological and non-physiological factors [30]. Physiological factors include differences in disease presentation and body size. Women often present with lower model for end-stage liver disease score due to lower creatinine levels and muscle mass, which may underestimate disease severity and reduce priority for transplantation. Recent scoring improvements aim to mitigate this bias [38]. Patient body size also plays a role, as shorter individuals face higher wait-list mortality because size-matched donor livers are less frequently available and smaller organs are often prioritized for pediatric recipients. Additionally, non-physiological factors, like referral patterns, contribute to this gap. Men, who more commonly present with indications for transplantation, are more likely to have an early referral to transplant centers [39, 40]. The impact of sex mismatch on transplant outcome is still less well understood. It is known that women are more often donors than recipients, and the female-donated liver to male patients seems to have the worst prognosis [41].

We know that HCC predominantly affects males with an incidence three to four times higher compared to women, depending on ethnicity, etiology, age, and other factors [27, 30, 42]. However, the frequency of MASLD-related HCC is significantly higher in women, suggesting that females are more susceptible to metabolism-associated liver damage [35, 42]. Our recent work proposed that chronic depletion of cholesterol synthesis in a mouse model is a possible metabolic trigger for sex-based liver disease progression, leading to female prevalent HCC in the aging mice [14].

## Factors associated with sex differences in MASLD and HCC

**Fig. 2:**
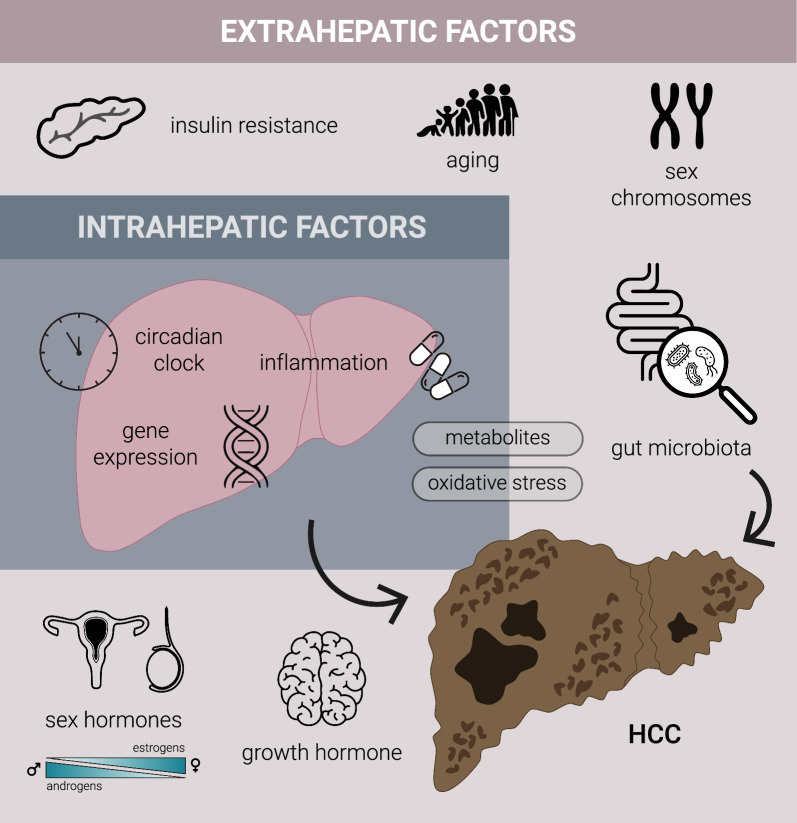
Multiple factors, including extra- and intrahepatic, contribute to metabolic-related hepatocarcinogenesis. Hepatic sexual dimorphism emerges from a complex interplay of intra- and extrahepatic factors including sex hormones, growth hormone, sex chromosome complement, ageing, metabolic and inflammatory cues, oxidative stress, gut microbiota, and circadian regulation. The regulation of sex-dependent metabolic pathways in the liver depends to a large extent on growth and sex hormones. The concentration of female estrogens are three times higher than that of androgenes and peak in accordance with the monthly estrus cycle. The growth hormone also has a monthly peak, which is due to the feedback of the ER to the growth hormone. In contrast, the concentrations of male androgens are 10 times higher than those of estrogens, while the concentration of growth hormone shows daily fluctuations over a 24-h period. *The figure was created in Adobe Illustrator*

Sex-related differences in the liver are defined by multiple factors, including the action of sex hormones, growth hormone (GH), the influence of sex chromosomes and their interactions [25], and also by differential influence of multiple genes, gene products, metabolites, the effect of insulin in light of resistance, the inflammation and oxidative stress, gut microbiota and also the circadian regulation of these processes that, on top, depend on age [14, 17, 18] (Fig. 2).

### Role of sex hormones and chromosomes

Sex-based differences in hepatic processes and disorders are undoubtedly guided by sex hormones [43]. While the development of breast cancer is modulated by estrogens and prostate cancer by androgens, the development of HCC may depend on androgens, estrogens or both hormone types [44, 45]. Androgens are generally considered to cause HCC, while estrogens should have a protective role [45, 46]. Some older studies show that oral contraception containing higher concentrations of synthetic estrogens and/or progestins may result in a higher incidence of precancerous liver stages, such as focal nodular hepatic hyperplasia, hepatic hemangioma, and hepatocellular adenoma [47–49]. In addition to hormones, environmental pollutants such as 2,3,7,8-tetrachlorodibenzo-p-dioxin (TCDD) can interact with estrogen signaling [50, 51] and initiate HCC development in rodent models [52].

**Estrogens** bind to estrogen receptors (ERs), including the nuclear receptor isoforms ERα and ERβ and the membrane-bound G protein-coupled estrogen receptor (GPER). Both, nuclear and membrane ERs are expressed in hepatocytes in mice and humans of both sexes, but at lower level in comparison to reproductive organs [16, 53]. Zhou et al*.* showed higher expression of ERα than ERβ in hepatocytes and hepatic stellate cells in rats [54]. In women, liver expression of ERα is stable even during aging, while changes in receptor expression may occur during puberty or hormone replacement therapy [53]. It is well established that estrogen hormones and receptors are involved in the sex bias of MASLD pathogenesis. In ovariectomized (OVX) female mice, a high fructose diet enhanced liver cell destruction, macrophage accumulation and progression of fibrosis and estrogen replacement improves insulin sensitivity [55]. The exact mechanism of GPER mediated by estrogens in MASLD is not yet fully understood [17].

When estradiol (E2) acts on ER, the receptor-ligand complex is transferred to the nucleus, where it can bind to specific genomic sequences and causes gene transcription or silencing. Estrogen responsive elements (EREs) are present in liver promoters and enhancers. The genetic analyzes show sex bias in the expressions of over 1000 hepatic genes in humans. Among them more than 40 sex-related genes belong to lipid metabolism [56–58]. Alterations in expression of genes regulated by estrogens associate with an increased risk for metabolic liver diseases: *LDLR* [59, 60], *APOA5* [61] and *ABCA1* primarily in women, and *LIPC* in men [58]. Li et al*.* have shown that improper co-regulation of transcription factors FOXA1/2 and ERα affect the expression of several genes involved in hepatocarcinogenesis. The induction of carcinogenesis by diethylnitrosamine (DEN) in mice with hepatocyte *Foxa1/2* deletion caused altered expression of *BTG1, FGL1, ABCC4,* and *PPM1L,* where individual variants at *FOXA2* binding sites were associated with an increased incidence of HCC in women [62]. A recent study showed six sex-specific *Esr1* transcripts, indicating sex specific differential splicing implicated in hepatocarcinogenesis. Downregulated *Fmo3* was identified as important lipid metabolism-related marker [63].

**Androgens**, such as testosterone and dihydrotestosterone (DHT), act through the androgen receptor (AR), a nuclear transcription factor expressed in the liver of both humans and rodents [53], with sex and age dependent expression levels [64]. For instance, hepatic AR is almost undetectable in aged and prepubertal rats compared to the postpubertal stage [65]. In animal models, the role of AR in lipid metabolism is well established: mice lacking AR develop more severe steatosis, insulin resistance, and lipid accumulation when challenged with a high-fat diet [66, 67]. Decreased PPARα (Peroxisome Proliferator Activated Receptor Alpha, NR1C1) expression represents a possible mechanism for steatosis with insulin and leptin (LEP) resistance in these mice [68]. AR can paradoxically limit metastasis in later tumor stages, indicating that its effects change as the disease progresses [67]. In humans, men have a higher HCC incidence than women, which correlates with higher AR expression and androgen activity. In contrast, human data on circulating androgens and MASLD are inconsistent: some clinical studies associate higher androgen levels with MASLD, while others report protective effects. These discrepancies likely reflect heterogeneous study populations, such as women with polycystic ovary syndrome (PCOS), anabolic steroid users, and hypogonadal men [53, 67]. Human HCC frequently expresses AR, yet anti-androgen therapies have not demonstrated clear clinical benefit, highlighting the complexity of this pathway [69].

The role of androgens in women who have lower basal androgen levels compared to men has not yet been elucidated. Hyperandrogenism in women can lead to PCOS and poses a higher risk of developing MASLD. This was not associated with insulin resistance or obesity but with hepatotoxic liver damage [70].

**Growth hormone (GH)** effects on diverse expression of genes in the liver and adipose tissue. It regulates lipid and carbohydrate metabolism and the metabolism of xenobiotics and steroid hormones [71] and is crucial for liver disease progression by controlling inflammation and liver regeneration [55]. GH binds to the hepatic transmembrane growth hormone receptor (GHR), activating the transcription factor STAT5, which regulates a variety of sex-related genes. Both GH levels and secretion patterns are important in liver metabolism and fibrosis [72, 73]. Low GH levels, as seen in obesity or GH deficiency models, reduce STAT5 activity in both sexes, promoting hepatocyte lipid accumulation and MASLD [55]. On the other hand, pulsatile GH secretion in males makes STAT5 activation dynamics more sensitive to disruptions, while continuous GH in females maintains moderate but persistent STAT5 activation [74, 75]. These sex-specific GH–STAT5 dynamics regulate different subsets of transcription factors and likely underlie differences in MASLD susceptibility, which is higher in males than in females [76]. Estrogens can provide additional protective support by directly stimulating hepatocytes to increase STAT5 activation through both GH-dependent and GH-independent mechanisms [73].

Liver sex-based dimorphism is traditionally attributed to the dramatic effects of hormones. However, human studies remain heterogeneous, with inconsistencies likely arising from differences in study design, population characteristics, and lack of stratification by menopausal status. Therefore, the current evidence base remains fragmented, underscoring the need for harmonized, sex-stratified clinical studies.

Meanwhile, genetic differences in sex chromosomes may also be associated with metabolic dysregulations [58, 77, 78]. Generally, functional variations in X-linked genes have more impact on men compared to women [79]. Marrone et al*.* showed high prevalence of MASLD in Klinefelter Syndrome (XXY) patients [80]. Zhang et al*.* and Lv et al*.* also exposed that sex-determining region on the chromosome Y (SRY) may correlate with sex bias in HCC [81].

Furthermore, sex-biased genes enriched in sex chromosomes and accompanied with sex-biased DNA methylation associate with the cell cycle, metabolism pathways, immune response, DNA repair and p53 pathways [82]. The research analysis of HCC samples in The Cancer Genome Atlas (TCGA) database, including normal and neoplastic tissue for each sex, reported that pathways related to lipid metabolism were significantly deregulated only in male patients [83]. Decreased expression of the *DAX1* and its effect on the Wnt/β-catenin signaling pathway in hepatocarcinogenesis has been shown on X chromosome in several XC cell lines [84].

### Aging as a risk factor for HCC in males and in post-menopausal females

The link between sex and age is another challenging factor due to changed hormonal effects. The distinction in individual reproductive periods, such as the menstrual cycle, pregnancy, menopause or post menopause, is crucial in women's incidence of hepatic diseases [28]. Recent data indicate a trend in increasing MASLD prevalence among postmenopausal women, especially those between 60 and 69 years of age [26, 85]. Menopause associates with accelerated development of hepatic fibrosis and increased risk of HCC [86–88], although the percentage of HCC incidence in females remains lower compared to males. The increased incidence of liver disease in older females is attributed to a drop in estrogen levels. The reason is in increased activity of estrogen dependent IL-6/STAT pathway, which is responsible for inflammatory processes, inducing hepatocyte damage and cell proliferation [88]. The report from 2016 showed that duration of estrogen deficiency in postmenopausal status, age at menopause and time from menopause appear to determine the fibrosis risk among postmenopausal women with MASLD [89]. Two views about age dependent effects on MASLD development in aging women was introduced. In one the age was proposed as an independent predictor only in women, while the other study showed age as a risk factor for MASLD only in premenopausal women, but not in menopausal women [90]. Additional findings indicate that older women with MASLD are at higher risk for mortality than men [85].

During the childbearing age, E2 is formed mainly in the ovaries, and the concentration of E2 is about five times higher than in men. After menopause, the production of estrogens takes place in peripheral tissues, such as adipose tissue, adrenal gland, vascular endothelium, bone, and smooth muscle. E2 is produced from testosterone and estrone from androstenedione. Surprisingly, estrogen levels in postmenopausal women are comparable to men of the same age group [53]. Some studies show that E2 concentration can be even lower in women after menopause than in men [91]. Deregulated metabolic processes in hepatocytes contribute to development of metabolism-associated HCC, and the difference between sexes seems to be associated with disturbed steroid homeostasis in the adipocytes. In postmenopausal women and older men, reduced energy expenditure occurs with increased visceral fat, body weight, triglycerides, and cholesterol [92]. Increased visceral fat has been linked to HCC development [93], recurrence [94, 95], and decreased survival [96]. Mechanistically, visceral fat accumulation alters circulating lipids, increases reactive oxygen species (ROS), and affects adipokine and hormone secretion [97]. Adipocyte hypertrophy can lead to local hypoxia and chronic inflammation, elevating pro-inflammatory cytokines (IL-6, TNFα), recruiting M1 macrophages, and promoting insulin resistance, collectively creating a tumorigenic microenvironment. The *ADIPOQ* and *LEP* in adipocyte processes may be a key connection between visceral fat and hepatocarcinogenesis [98]. Notably, ADIPOQ decreases in men with obesity and during puberty, contributing to their higher liver cancer risk [99]. A possible explanation for decreased ADIPOQ levels could be associated with the proposed novel marker for MASLD, miR-375, that was found increased in liver sections of HFD-fed mice and in palmitic-acid treated HepG2 cells [100]. Lei et al. showed that the miR‑375 targets mRNA of the ADIPOQ receptor (*AdipoR2*) in hepatocytes, reducing its expression and blunting ADIPOQ-mediated anti-inflammatory signaling. Notably, restoring AdipoR2 lowers inflammatory cytokines, conditions that support higher ADIPOQ levels. LEP has a dual role in MASLD models. It physiologically reduces hepatic fat accumulation, while in MASLD and obesity its elevated levels lead to LEP resistance, promoting inflammation, insulin resistance, and hepatic fibrosis through pathways such as JAK2/STAT3 and SOCS3. Clinically, circulating LEP levels in MASLD are generally normal or elevated compared with controls, but associations with severity are inconsistent [101, 102]. Hossain et al*.* also reported that women had significantly higher serum LEP levels than man, along with other sex-specific patterns linked to MASLD [103]. Additional evidence points to other age-related modifiers. For example, elevated uric acid increased MASLD risk in Korean postmenopausal but not premenopausal women, underscoring the hormonal dependence of metabolic injury in aging females [104].

Overall, aging increases sex-specific vulnerabilities to MASLD and HCC, primarly because postmenopausal estrogen loss in women and earlier visceral fat accumulation in men drive distinct metabolic and inflammatory pathways.

### Linking sex differences and the liver drug metabolism

Hepatic metabolism generally depends on the sex. Sex-based are both metabolism of endobiotics, representing compounds produced by the body, and also of xenobiotics—the chemical substances that are not naturally produced by the organism, such as drugs, environmental pollutants, food additives, etc*.* Sex differences in the expression of hepatic drug metabolizing enzymes (DME) are documented for genes/enzymes of the primary metabolism (CYP450s), secondary metabolism (i.e. sulfotransferases, glutathione transferases) and the transporters [105]. Experiments with male and female primary hepatocytes further demonstrate sex-specific responses to hepatotoxicants, with female hepatocytes generally showing greater sensitivity to certain drugs [106]. Similarly, clinical studies show that women experience adverse drug reactions almost twice as often as men, largely due to sex-biased pharmacokinetics [107]. Recognizing these differences is therefore crucial for optimizing pharmacotherapy and reducing the risk of adverse reactions, particularly in patient populations with altered DME expression, as observed in both rodent and human samples with MASLD and MASLD-associated metabolic comorbidities [108].

One primary biological factor contributing to sex differences in drug metabolism is the differential expression of CYP450 enzymes, both in human and in animal models [109, 110]. Particularly important is the major human DME CYP3A4 [111]. Many drugs that are CYP3A4 substrates show higher clearance in women than in men, a difference associated with increased hepatic *CYP3A4* expression in females. This has been demonstrated for verapamil, a calcium channel blocker, and zolpidem, a hypnotic agent used to treat insomnia [111, 112]. However, this is not supported by all studies which might be due to the large range of inter-individual differences in human hepatic CYP3A4 protein levels (10- to 20-fold) in human liver tissue banks [113].

Another layer of complexity in drug metabolism may be the sex differences in gut microbiome composition [32, 114]. The gut microbiome, a community of microorganisms naturally residing in the intestine, has recently emerged as a key factor in maintaining human health and influencing the pathophysiology of various diseases. Consequently, disruptions to its composition or diversity have been linked to the onset and progression of liver cirrhosis and MASLD. In particular, the loss of beneficial bacteria that produce short-chain fatty acids, along with the expansion of ethanol- and secondary bile acid–producing bacteria, may promote gut barrier dysfunction, hepatic inflammation, and fibrosis [115], and consequently affect hepatic drug metabolism. Additionally, the gut microbiome harbors enzymes that can modify drug metabolism, altering both efficacy and toxicity. For instance, *Eggerthella lenta* can inactivate digoxin, a cardiac medication, by converting it into an inactive metabolite [116]. Conversely, the gut microbiome can activate prodrugs and enhance their pharmacological activity [117]. Importantly, several microbiome–drug interactions show emerging sex specificity. For example, the level of β-glucuronidase in gut metagenomes was higher in males than in females, providing a potential explanation for sex-based differences in efficacy and toxicity for several drugs [118]. These examples illustrate that microbiome-encoded enzymes do not act uniformly across sexes but interact with host biology to modify pharmacokinetics and toxicity profiles.

Sex hormones, such as testosterone and E2 can shape the gut microbiome. Individuals with higher serum levels of sex hormones exhibit greater diversity in gut microbiota [119]. A potential role of the gut microbiome in regulating sexually dimorphic opioid response in humans was discussed in a recent review paper [120]. It was concluded that both sex hormones and opioids modulate the composition and activity of the gut microbiome and that the effects of chronic opioid exposure on sex hormone regulation could in turn influence microbiome in a sex-dependent manner. These bidirectional effects suggest that changes in sex hormone levels, whether physiological, disease-related, or medication-induced, may shift microbial drug-metabolizing capacity in opposite directions in women and men, thereby altering exposure to active drug metabolites [121, 122].

Interestingly, in rodent models, puberty-induced hormonal changes result in a less diverse gut microbiome in males compared to females [123]. This interaction between sex and the gut microbiome has significant implications for personalized medicine. Several studies have proposed probiotics as adjunct therapy for MASLD [124]. Although these studies did not consider sex-specific effects, probiotics could potentially be tailored for sex-specific interventions. Variations in microbiome composition and enzyme expression can lead to differences in drug efficacy and adverse reactions between men and women. Recognizing these differences is essential for developing sex-specific dosing regimens and therapeutic strategies.

### Sex specific circadian circuits and metabolic liver diseases

Being the fundamental metabolic organ, the liver controls whole-body homeostasis. More than half of liver metabolites show circadian rhythmicity. This is majorly under central and other peripheral clock control, while approximately 10% of the rhythmic transcripts are autonomously expressed [125]. Based on the tight circadian coordination of liver metabolism, it is not surprising that disruption of the circadian clock may lead to the development of diverse metabolic diseases and aggravated liver pathologies. Behavior-metabolism misalignment as in the case of social jet lag and shift-work is becoming a dominant risk factor for metabolic diseases [126]. Core clock genes maintain 24-h circadian rhythms through a transcriptional-translational feedback loop: CLOCK and BMAL1 form a heterodimeric transcriptional activator complex that drives rhythmic expression of *Per*, *Cry,* and other downstream genes. PER1/2 and CRY1/2 proteins accumulate and feedback to inhibit CLOCK-BMAL1 activity, generating self-sustained circadian oscillations [127]. Mouse models manipulating these components demonstrate their essential roles in metabolic regulation. PER2-deficient mice exhibit with altered lipid metabolism [128], while *Bmal1*^*−/−*^ and *Clock*^*−/−*^ mutant mice develop hyperlipidemia and hepatic steatosis [129, 130]. The latter is also seen as a result of Rev-erbα and Rev-erbβ deficiency [131]. Liver-specific deletion of *Bmal1* in mice can lead to the accumulation of ROS, resulting in increased mRNA methylation and downregulation of PPARα, which affects liver metabolism. Not only disruption of the clock genes but also chronic circadian disruption or/and ablation of crucial metabolic transcriptional co-regulators such as SRC-2 (Steroid Receptor Coactivator-2) can affect metabolic homeostasis. Altered behavior and metabolic homeostasis in *Src2*^*−/−*^ mice lead to MASLD, MASH, and HCC [126]. The circadian clock also plays a role in the modulation of bile acid metabolism where misalignment with feeding and fasting cycle leads to cholestatic disease [132], and is also engaged in glucose homeostasis, where CRY1 is involved in controlling hepatic gluconeogenesis [133]. It is important to mention that the circadian studies of metabolic liver diseases in animals have still a considerable male bias. However, sexual dimorphism in circadian physiology has been lately gaining more recognition since the circadian output parameters, such as hepatic drug metabolism, differ between females and males [134]. There is a sex difference also in the suprachiasmatic nucleus (SCN) morphology and signaling to and from SCN which may contribute to sexual dimorphism of clock-controlled processes [135].

HCC is the predominant type of liver malignancy and one of the most important causes of cancer-related deaths worldwide. It is primarily associated with chronic Hepatitis B Virus (HBV) and HCV infection, alcoholic cirrhosis, and aflatoxins. However, growing MASLD, obesity, metabolic syndrome as well as circadian clock disruption are also related to HCC [101, 136, 137]. A more detailed description of the biological clock relationship with liver cancer is described elsewhere [126]. Different clock gene knock-out mouse models revealed that genetic and epigenetic alterations of the core clock genes can increase early MASLD progression to MASH, fibrosis, and HCC. Bur et al*.* [138] proposed *Cry1* and *Cry2* as genetic determinants of sexual dimorphism of the liver metabolism. Male mice with knock-out of *Cry1* and *Cry2* genes expressed several sex-specific liver products, whose levels almost reached those in females. For example, *Cyp2b9* a predominantly female liver gene, was significantly up-regulated in the male knockouts and the feminized liver expression profile has been reversed by hormonal injections mimicking the male GH release pattern.

### The role of nuclear receptors in sex-dependent liver metabolism

Metabolic homeostasis, circadian timing, and the action of nuclear hormone receptors are closely interconnected. Some nuclear receptors participate in core circadian clock mechanisms (e.g. REV-ERB, RORs). Disruption of circadian rhythm leads to overexpression of the metabolism-associated nuclear receptor Constitutive Androstane Receptor (CAR; *NR1I3*), which drives the progression of MASLD toward HCC [139]. A study conducted by Lu et al*.* [140] demonstrated circadian variation and sex differences in hepatic metabolism genes and their corresponding receptors with higher levels of *Cyp* transcripts in female mice. These include *Cyp* targets of Aryl Hydrocarbon Receptor (AhR: *Cyp1a1, Cyp1a2*—metabolic activation of carcinogens), PPARα (*Cyp4a10, Cyp4a14*—lipid and energy metabolism), CAR (*Cyp2a4, Cyp2b10*), Farnesoid X Receptor (FXR; *NR1H4*: *Cyp7a1* and *Cyp27a1*), and Pregnane X Receptor (PXR; *NR1I2*: *Cyp3a11* and *Cyp3a44I*). In contrast, the male-predominant representative with circadian variation in mice is *Cyp7b1*. Circadian and sex variation of *Cyp* genes expression is most apparent in rodents (mice, rats), but also present in human livers. The most abundant human liver CYP450 enzyme is CYP3A4, which assists in the oxidation of bile acids and steroids and accounts for about 50% of oxidative drug metabolism. The metabolic activity of CYP3A4 is sexually dimorphic, which might explain the faster clearance of various drugs in women. Biological clock inevitably controls expression of CYP enzymes. Disruption of the clock gene expression may alter nuclear receptor signaling pathways, impact circadian expression of *CYP*s and may also lead to manifestations of metabolic syndrome [140]. Most importantly, understanding sex differences and variation of CYP enzymes rhythmicity can serve as the molecular basis for appropriate timing of drug administration, affecting efficacy and toxicity, along with improving chronotherapy [140, 141].

Upon binding to oxysterols that are expressed in a circadian manner, the metabolic sensor, nuclear receptor LXRα (Liver X Receptor alpha, *NR1H3*) regulates transcription of genes crucial for glucose, cholesterol, and fatty acids homeostasis [142]. Consequently, the glucocorticoid hormones, which play a pivotal role in metabolic homeostasis, show sexually dimorphic circadian patterns of secretion. LXRα also greatly impacts resetting the clock properties in the male mice. The glucocorticoid hormones are also potent synchronizers of the peripheral circadian clocks [142]. In *Lxrα*^*−/−*^ females sex-dependent alteration of the insulin and LEP oscillation were observed, which could be a result of a changed corticosterone profile. *Lxrα*^*−/−*^ males showed a lowered amplitude of plasma glucose oscillation which may be at least partially a result of altered *Pepck* stimulation by locally produced glucocorticoids. Therefore, LXRα is required for normoglycemia as well as for the normal daily glucose oscillation, proposing LXRα as a determinant of sexually dimorphic circadian patterns of key physiological parameters [142].

Peripheral clocks can be reset by other cues and not only by SCN derived signals. One of those regulators is a feeding-fasting cycle, since food is usually provided in a periodic manner. Calorie restriction (CR) increases lifespan and delays aging but also affects many other physiological systems (e.g. hormone levels, stress resistance, cancer incidence, glucose homeostasis) with favorable health effects [135]. Restricted feeding influences daily rhythms of several clock genes in mammals and suggested that beneficial effects of CR are sex-dependent [135, 143]. For example, the sex of the mice affects the outcome of CR on liver metabolism and gene expression. Astafev et al*.* [135] reported that a 30% CR diet affects the liver circadian clock gene expression in female and male mice and the expression of *Cry1, Cry2, Rorγ,* and *Rev-erbα* clock genes significantly differed between both sexes. Accumulating data indicate that sex is an important element in many physiological functions and an important modulator of circadian rhythms in gene expression and their response to CR [135].

### Epigenetic regulation of sex-specific hepatic gene expression

Another key factor influencing liver metabolism and HCC is epigenetics, encompassing global DNA hypomethylation of oncogenes, hypermethylation of tumor suppressors, histone modifications that alter chromatin accessibility, chromatin-loop rearrangements facilitating enhancer–promoter oncogenic interactions, and post-transcriptional regulation via microRNAs and RNA modifications [144]. Epigenetic mechanisms have also been shown to mediate sex differences in liver physiology, affecting both normal liver metabolism and disease susceptibility, as recently reviewed by Ye et al. [145].

Among epigenetic layers, DNA methylation is by far the most extensively studied in the context of sex differences in liver disease. In HCC, many tumor suppressor genes and regulatory loci exhibit sex-dependent DNA methylation patterns, including genes involved in cell-cycle regulation (e.g.* CDKL2*, *CDKN2A*) and repetitive elements (e.g. LINE1), which influence genomic stability [145]. For instance, *CDKL2* promoter hypermethylation, which is strongly linked to reduced gene expression, occurs significantly more often in women (48.0%) than in men (37.7%) [146]. In contrast, findings for *CDKN2A* promotor methylation (encoding p16^INK4A^, endogenous CDK inhibitor) are inconsistent [147, 148]. Importantly, one study showed that tumors with promoter methylation were more sensitive to the CDK4/6 inhibitor Palbociclib [149]. Validation of sex-based differences would provide a rationale for sex-selective therapeutic strategies, tailoring CDK4/6 inhibitor use according to sex-specific epigenetic patterns.

Other epigenetic mechanisms also contribute to hepatic gene regulation, but systematic analyses stratified by sex remain scarce. A recent study on mice demonstrated that maternal obesity induced sex-specific alterations in the repressive histone mark H3K9me3 in offspring livers, which were accompanied by marked male–female differences in gene expression, particularly in metabolic and stress-response pathways [150].

The differential patterns of methylation likely reflect the interplay of sex chromosomes, sex hormones, and tissue-specific chromatin regulation. Because many epigenetic alterations are reversible, targeting these mechanisms offers a potential sex-informed approach in HCC. Interventions designed to modulate male- or female-biased oncogenic or tumor-suppressive pathways could mitigate sex-dependent genomic instability and therapy resistance [151], while minimizing off-target toxicity in non-tumor tissues.

## Is liver fibrosis a sex and age dependent phenomenon?

Liver fibrosis is one of the hallmarks of liver disease progression and its resolution is one of the end-points of clinical trials [20]. Fibrogenesis is a wound healing response to injury and several cell types and signaling pathways are involved [152]. Studies have consistently confirmed that men have a higher risk for development of fibrosis compared to pre-menopausal women independently of the body mass index (BMI), while after the onset of menopause the risk evens up [153–156]. The male sex was associated with liver fibrosis already in the adolescent population [157]. A meta-analysis of 54 studies reporting the sex-stratified MASLD confirmed that men have a higher risk to develop MASLD, while the prevalence of advanced fibrosis was higher in over 50-year-old women in comparison to men [158]. This indicates that once women have established MASLD, they have a higher risk for the development of advanced fibrosis, especially those older than 50 years. In postmenopausal women, the duration of the estrogen deficiency (therefore age) is the key factor determining the severity of liver fibrosis [89]. In addition, a large cohort study of MASLD patients (N = 761,403) has shown that women have a higher incidence of liver-related adverse events and cirrhosis, while men have a higher incidence of liver decompensation, HCC and extrahepatic complications such as chronic kidney disease, cardiovascular disease, and non–sex-specific cancers [159]. Different animal models have also confirmed that fibrosis, induced by toxins or diet, is a male-prevalent process [160–162]. In OVX female mice, fibrosis severity also increases, but treatment with E2 or other ER agonists mitigates this effect [160, 163–166]. In women, however, estrogen replacement therapy was not yet confirmed to have a significant beneficial effect in reducing the fibrosis risk, indicating that other factors are important [89, 155].

Not only are female menopause and low estrogen levels associated with fibrosis, also the male menopause and lower testosterone are associated with worse MASLD [167]. Lower testosterone in combination with high sex hormone-binding globulin concentration was associated with higher liver fibrosis in middle-aged men [168]. Interestingly, in women, the effect of high testosterone was associated with higher odds of MASLD [167]. This pattern is evident across ages: in adolescent cohorts, higher testosterone is protective in boys but detrimental in girls [169], and in young women it correlates with increased the fibrosis risk and early progression toward MASH [170].

The association of different risk factors with the degree of liver fibrosis is also sex-dependent. For example, BMI has a different effect on the prevalence of advanced fibrosis depending on sex and age [171]. The size and the position of fat depots are associated with fibrosis severity depending on sex and menopausal stage [172]. In type 2 diabetic cohort with an average age of over 50 years old, female sex was associated with advanced fibrosis [173]. After liver transplantation, the male sex was associated with fibrosis and MASLD development [174]. Sex does not only affect the development of fibrosis but also the associated factors and fibrotic scores [175]. When comparing different non-invasive biomarkers of fibrosis, men had significantly higher FIB-4, FLI and APRI scores than women, while no sex difference was observed in the NFS score [176, 177]. These differences may reflect how each score incorporates factors such as liver enzymes, platelet count, metabolic parameters, and fat distribution, which are influenced differently by sex, rather than indicating actual differences in fibrosis severity. Similarly, the state of steatosis, inflammation and hepatocyte injury is not always consistent with a degree of fibrosis. A cross-sectional study in a biopsy-proven MASLD cohort showed higher hepatocyte injury and inflammation in premenopausal females, but a lower stage of liver fibrosis in comparison to post-menopausal females and men [178]. This further confirms that the impact of sex on fibrosis in the context of MASLD development and progression is multifaceted.

Genes and pathways involved in sex-specific fibrogenesis are still unresolved, but some indications already exist. There is evidence that estrogen has an effect on hepatic stellate cells activation and differentiation, which is essential for fibrogenesis [179]. A recent study in mice identified the PPARα co-expression network as sexually dimorphic in mice [180]. This was confirmed in a human MASLD cohort, where genes correlating to *PPARα* expression were more abundant in men than women. Further studies in mice confirmed that PPARα contributes to the sexually dimorphic response to dietary challenges in the liver and pemafibrate, a PPARα agonist tested for the treatment of MASLD [180]. Interestingly, in hepatocyte-specific *Pparα* knockout mice, the dietary challenge induced worse fibrosis in female mice. However, PPARα is the main regulator of lipid homeostasis in the liver and was proposed as the sexually dimorphic target in MASLD. The level of liver pyruvate kinase is regulated by testosterone and is strongly associated with MASLD in men, but not in women [181]. Silencing the liver pyruvate kinase attenuated the diet-induced liver fibrosis in males. Diet-induced MASLD in middle-aged mice also resulted in male-dominant development of fibrosis, while a similar degree of steatosis was present [182]. In this study, PCSK9 (Proprotein Convertase Subtilisin Kexin Type 9) and IL-1β (Interleukin 1 Beta) were identified as contributors to sex-specific changes in MASH transition. Female liver-specific *Ppargc1a* (Peroxisome Proliferative Activated Receptor, Gamma, Coactivator 1 Alpha) heterozygous mice challenged with high-fat high-fructose diet, developed worse fibrosis than males [183]. PPARGC1A is a partner of ER and this partnership is essential for estrogen-dependent response to oxidative stress. More severe fibrosis in male wild type SHRSP5/Dmcr rats on high fat-cholesterol-cholic acid diet was associated with sex-specific dysregulation of bile acid metabolism [184]. In males, bile acid detoxification-related enzymes such as glucuronosyltransferase (UGT) and sulfotransferase (SULT) and their regulators the nuclear receptors CAR and PXR were strongly suppressed, while only slightly in females.

Sex and menopause modify also the effect of genetic variations on the development of fibrosis in MASLD. A significant interaction was found with sex for 10 loci in genes *KCNIP4* (Potassium Voltage-Gated Channel Interacting Protein 4), *PSORS1C1* (Psoriasis Susceptibility 1 Candidate 1), *KLHL8* (Kelch-Like Family Member 8), *GLRA1* (Glycine Receptor Alpha 1), *NOTCH2* (Notch Receptor 2), and *PRKCH* (Protein Kinase C Eta), while four SNPs were intergenic [185]. An rs72613567-(-/A) + (A/A) genotype in the *HSD17B13* gene was associated with a lower risk of developing fibrosis and was protective in subgroups of patients with a *PNPLA3* rs738409-(CC) genotype, BMI over 35, age over 45, with diabetes type 2 and women [186]. As the liver is a highly sex dimorphic organ, the same genome could, therefore, result in a different disease severity due to sex differences in gene expression, exon usage and hormonal status.

Despite clear sex differences in fibrosis biology, very few clinical studies evaluated the sex-specific response to therapeutic strategies [187]. Weight loss appears more effective in improving fibrosis in men, whereas women require greater weight reduction to achieve similar histologic benefit (mean age of the cohort was 48.5 ± 9.6 years) [188]. The cross-sectional study of type 2 diabetes patients with MASLD and fibrosis on statins, revealed that statin use is associated with lower odds of advanced fibrosis and that this association was stronger in women [189]. Lower choline intake was associated with worse fibrosis in postmenopausal women, but not in men or pre-menopausal women [190].

In summary, liver fibrosis is sex- and age-dependent, with men generally developing fibrosis earlier in life, while older and postmenopausal women have a higher risk for advanced fibrosis. However, some cohorts show no association or even contradictory trends depending on age, BMI, or genetic background. These discrepancies indicate that sex alone does not fully explain fibrosis trajectories and must be interpreted in the context of interacting metabolic, inflammatory, and genetic factors. The lack of sex-specific mechanistic studies in humans further limits causal inference.

## Animal models of metabolic and sex-related HCC: are we moving in the right direction?

Diagnostic and therapeutic options in HCC patients are limited [191, 192]. Therefore, laboratory animal models still play an important role in basic and translational studies of liver pathogenesis. Despite their abundance and extraordinary use, not all animal models are suitable for all MASLD research areas, especially when assessing sex differences and sex/age interaction [18, 193, 194]. Among animal models, a majority of studies have been performed on mice. Mice represent predictive models for understanding the development of metabolic-related HCC, but with awareness of limitations in their use. Generally, MASH is difficult to model in mice because most models only recapitulate some MASH characteristics. Mice do not develop hepatocyte ballooning and cytoplasmic inclusions, termed Mallory-Denk-Bodies [195]. Besides, several monogenic human SNPs have been associated with MASH, but many rodent models engineered with these SNPs do not present with the MASH phenotype [196].

The laboratory mouse models are divided into xenograft models, chemically and/or diet- and virus-induced, and genetically modified models (GEMs) [193, 197–199]. GEMs with knocked out *Mdr2* (*ABCB4*) and *Aox* (Aldehyde Oxidase), respectively, are widely used in studies of progressed liver diseases induced by metabolic disorders. *Mdr2*^*−/−*^ mice have been a subject of intensive research on cholestasis, fibrosis, and carcinogenesis in humans. MDR2 is a flippase that regulates bile secretion in phospholipid micelles, along with bile acids and cholesterol. In *Mdr2*^*−/−*^ mice, the pathways associated with cell proliferation, oxidative stress, and lipid metabolism occur markedly in the pre-cancerous stage [200]. *Aox*^*−/−*^ mice have inhibited acetyl-CoA oxidase activity. An enzyme activity inhibition results in liver steatosis development, which leads to activation of PPAR and is manifested in the presence of liver adenomas and carcinomas [201]. Additionally, *Lep* knock out mice spontaneously develop steatosis and HCC without inflammation or cirrhosis when are exposed to a HFD. A recent study by Meyer et al*.* [202] demonstrated sex-specific differences in body weight, fat distribution and liver tissue changes in mice fed a 'fast food' diet, while Arivazhagan et al*.* [203] found higher MASH scores in females than in males on a diet enriched with fructose, palmitate and cholesterol.

Glycine N-methyltransferase (GNMT) is the main enzyme in the catabolism of excess hepatic S-adenosylmethionine. Its gene expression in the liver is significantly lower in patients with risk for HCC development. It was shown that GNMT knockouts (*Gnmt*^*−/−*^) develop liver steatosis, fibrosis, and HCC at 16 months of age [204]. Interestingly, there is a considerable lack of mouse models of liver cirrhosis recapitulating features of the human disease. Only one GEM with *Mcrs1* deletion in hepatocytes causes degradation of bile acid transport, and bile acid flow with HCC development is described [205].

Species, genetic background/strain and sex determine morphologic and metabolic consequences in liver pathologies [206]. Claper and colleagues highlight that aged C57BL/6 J mouse liver represents an excellent microenvironment for MASH development and offering the opportunity to study disease in female mice. Aged C57BL/6 J mice on the Amaylin liver MASH diet at 30 weeks develop steatosis, steatohepatitis with fibrosis, and cirrhosis which closely mimics the etiology of the disease in humans [207]. Phenotypically, mice with hepatocyte-specific *Pten* knock out are similar to patients with MASH-associated HCC. In *Pten* mice, aged 74 to 78 weeks, tumors occur in 83% of males and 50% of females [198]. *MUP-uPA* mice on an HFD were represented as a promising genetic model of MASH-associated HCC. Mice express high levels of plasminogen urokinase activator in hepatocytes, resulting in activation of endoplasmic reticulum stress and liver damage leading to HCC. Unfortunately, the study was conducted only on male mice [208].

Interestingly, most diet-driven MASLD models rarely develop HCC. For studies of MASLD induced HCC have proposed a mixed diet model combining choline deficient diet and HFD and C57BL/6 mice feeding a choline-deficient L-amino-acid-defined diet (CDAA), respectively [195, 209]. Besides, tumor development in diet promoted models is strain dependent, with the strain DBA/2 J being the most exposed to hepatocarcinogenesis [210].

As mentioned, the incidence of HCC in women increases with aging when estrogen levels decline. It is necessary to be aware of the limitations of comparing current mouse models with menopausal women. There are specific differences in the hormonal cycle between female mice and women, making mice not a suitable model to mimic menopause in humans. The hormonal cycle in mice is defined as the estrus cycle, while in women is appointed as the menstrual cycle. The estrous cycle, consisting of the proestrus, estrus, metestrus, and diestrus phases, cyclically occurs for four to five days [211]. The cycle phases in mice have similar hormonal fluctuations in the ovaries as seen in the menstrual cycle of women, which last between 21 and 35 days [212, 213]. Estropause, an irregular estrous cycle, begins in mice during 13 and 14 months of age [214]. In women, menopause or hormonal imbalance due to ovarian failure, which reduces progesterone, estrogen, and testosterone in the blood, usually occurs after the age of 50 [212]. At 17 months of age, about 80% of female mice experience irregular cycles or go into the anestrus phase. The anestrus phase occurs in all mice by 25 months [214]. Mice enter the anestrus phase when the ovulation cycle stops. Still, low concentrations of sex hormones are present all the time due to the hypothalamic-pituitary-gonadal axis (HPG). Despite altered regulation of HPG, mice in anestrus still maintain low levels of E2 in the bloodstream, whereas, in postmenopausal women, blood levels of E2 and progesterone are deficient [211].

In aging mouse models, more appropriate, the menopause can be mimicked by surgically induced menopause or OVX. The main limitation of those models is that the ovaries removal in mice does not mimic the natural transition of perimenopause to menopause by gradually changing the concentration of hormones in the blood. In addition to estrogens, OVX also depletes other hormones (androgens, LH, GnRH, FSH), which play an important role in menopause and affect brain function [215].

An additional limitation of using mouse models for studies of sex dimorphism in metabolism-related hepatocarcinogenesis is their lack of primate-specific adrenal steroidogenesis. In humans, the *zona reticularis* of the adrenal cortex produces the androgen precursors DHEA and DHEA-S (and also 11β-hydroxyandrostenedione) that peripheral tissues, including the liver, can convert into active steroid hormones. This pathway becomes particularly relevant after menopause, when ovarian estrogen production declines and adrenal precursors become the main substrates for local steroid synthesis [216]. However, the influence of this intracrine conversion on the development of liver pathologies remains unclear.

In summary, it is crucial to select an appropriate model based on the objectives of the studies. While rodent models remain indispensable, they may not adequately reflect the sex-specific physiological features of human liver disease. A major limitation is their lack of human-like adrenal steroidogenesis, which is particularly relevant for modeling metabolic and carcinogenic processes in postmenopausal females. Rodents differ from humans also in their reproductive cycling, as the short estrous cycle cannot reproduce the human menstrual cycle. Mice have also a restricted ability to develop certain histopathological features of liver disease, such as hepatocyte ballooning and progressive fibrosis, which reduces the translational relevance of many MASLD and MASH models. These limitations are further amplified by the persistent male bias in metabolic and carcinogenesis studies of liver disease, which hinders identification of sex-specific mechanisms and limits the applicability of preclinical findings to humans.

### Summary box

**Table:** 

1. The liver is one of the most sexually dimorphic human somatic organs in terms of metabolic functions, with pronounced sex differences in xenobiotic metabolism, lipid homeostasis, and immune signaling
2. Hepatic sexual dimorphism results from the interplay of sex hormones (androgens, estrogens), growth hormone, sex chromosomes, ageing, insulin resistance, inflammation, oxidative stress, gut microbiota and circadian regulation
3. Sex-dependent differences in hepatic drug and lipid metabolism influence susceptibility to liver disease, therapeutic efficacy and the results of liver transplantations
4. Men are more susceptible to malignant liver tumours, cholangitis, primary sclerosis and hepatitis, while women are more likely to suffer from benign lesions, alcohol-related damage and autoimmune liver diseases. MASLD and MASH are more common in men and postmenopausal women
5. HCC is three to four times more common in men, while women are more susceptible to metabolic-associated liver damage, with the risk of HCC increasing in postmenopausal women
6. Estrogens generally have a protective effect against HCC and fibrosis. Their deficiency increases the risk of liver disease in postmenopausal women and men with low testosterone levels. An elevated testosterone level protects men from steatosis, but is associated with a higher risk of MASLD in women
7. The progression of fibrosis is modulated by both sex and age. The risk accelerates in women after menopause and may exceed the risk observed in men
8. Over 1,000 hepatic genes are expressed in a sex-specific manner, including more than 40 genes involved in lipid metabolism. Changes in hormone-regulated gene expression contribute to an increased risk of metabolic liver diseases
9. More than half of liver metabolites follow circadian rhythms that differ between the sexes. Disruption of the circadian clock can lead to metabolic diseases and exacerbation of liver pathologies
10. Sex-specific therapeutic responses are still poorly understood. Men benefit histologically more from weight loss, while women seem to benefit more from statin therapy
11. Animal models remain essential to elucidate sex-specific mechanisms in liver pathophysiology, but their translational value in the context of metabolic-related hepatocarcinogenesis is limited

## Conclusions and future directions

In summary, our review highlights that liver sexual dimorphism arises from a complex interplay between hormones, circadian signaling, genetic determinants, and even gut microbiome influences, all of which contribute to distinct metabolic profiles and different disease outcomes in men and women. Estrogens and androgens, together with growth hormone, modulate liver metabolism, inflammation, and fibrosis in a sex-specific manner. Aging and menopause amplify these disparities by altering hormone levels and metabolic homeostasis, particularly through changes in lipid handling, insulin sensitivity, and adipokine signaling. Clear sex differences in CYP450 expression, drug clearance, and microbiome-driven metabolite production also influence hepatic disease progression, therapeutic response, and toxicity. Circadian studies further demonstrate sex-dependent rhythmicity in nuclear receptor activity and metabolic gene expression, with clock disruption accelerating steatosis, fibrosis, and carcinogenesis.

Recent advances in RNA-seq and improved animal models have deepened our understanding of these processes. Despite this progress, several mechanistic questions remain insufficiently addressed, including how sex hormones interact with circadian and metabolic transcriptional networks, how chromosomal versus hormonal factors uniquely shape hepatocarcinogenesis, how the gut microbiome contributes to sex-specific differences in drug metabolism, and which molecular pathways account for the divergent fibrosis trajectories observed in men, premenopausal women, and postmenopausal women. Addressing these unresolved aspects will require coordinated multi-omics approaches, more humanized experimental systems, and rigorously designed sex-stratified clinical studies capable of disentangling these complex biological interactions. Such efforts will be critical for designing targeted therapies and optimizing clinical management to address the unique hepatic pathophysiological mechanisms in both sexes.

## Data Availability

No datasets were generated or analysed during the current study.

## Abbreviations

ABCA1: ATP binding cassette subfamily A member 1
ABCC4: ATP binding cassette subfamily c member 4
ADIPOQ: Adiponectin
AdipoR2: Adiponectin receptor 2
A-FABP: Adipocyte fatty acid-binding protein
AhR: Aryl hydrocarbon receptor
AIs: Artificial intelligence applications
AOX: Aldehyde oxidase
APOA5: Apolipoprotein A5
APRI: AST to platelet ratio index
AR: Androgen receptor
BMAL1: Basic helix-loop-helix ARNT like 1
BMI: Body mass index
BTG1: BTG anti-proliferation factor 1
CAR: Constitutive androstane receptor
CDAA: Choline-deficient L-amino-acid-defined diet
CDK: Cyclin-dependent kinase
CDKL2: Cyclin-dependent kinase-like 2
CDKN2A: Cyclin-dependent kinase inhibitor 2A
CLOCK: Clock circadian regulator
CR: Calorie restriction
CRY1: Cryptochrome circadian regulator 1
CYP450: Cytochrome P450
DAX1: Nuclear receptor subfamily 0 group B member 1
DEN: Diethylnitrosamine
DHEA: Dehydroepiandrosterone
DHEA-S: DHEA-sulfate
DHT: Dihydrotestosterone
DME: Drug metabolizing enzymes
E2: Estradiol
ER: Estrogen receptor
EREs: Estrogen responsive elements
ESR1: Estrogen receptor 1
F: Stage of fibrosis
FGL1: Fibrinogen like 1
FIB-4: Fibrosis-4
FLI: Fatty liver index
FMO3: Flavin containing dimethylaniline monoxygenase 3
FOXA1: Forkhead box A1
FOXA2: Forkhead box A2
FSH: Follicle-stimulating hormone
FXR: Farnesoid X receptor
GEM: Genetically modified models
GH: Growth hormone
GHR: Growth hormone receptor
GLRA1: Glycine receptor alpha 1
GNMT: Glycine N-methyltransferase
GnRH: Gonadotropin-releasing hormone
GPER: G Protein-coupled estrogen receptor
HBV: Hepatitis B virus
HCC: Hepatocellular carcinoma
HCV: Hepatitis C virus
HFD: High-fat-diet
HIF1: Hypoxia inducible factor 1
HPG: Hypothalamic-pituitary-gonadal axis
IL-1β: Interleukin 1 Beta
JAK: Janus kinase
KCNIP4: Potassium voltage-gated channel interacting protein 4
KLHL8: Kelch-like family member 8
LDLR: Low density lipoprotein receptor
LEP: Leptin
LH: Luteinizing hormone
LINE1: Long interspersed nuclear element-1
LIPC: Lipase C
LXR: Liver X receptor
MASH: Metabolic dysfunction-associated steatohepatitis
MASLD: Metabolic dysfunction-associated steatotic liver disease
MASL: Metabolic dysfunction-associated steatotic liver
MDR2: Multidrug resistance P-glycoprotein 2
MUP-uPA: Mice expressing transiently high amounts of urokinase plasminogen activator in hepatocytes
NAFLD: Non-alcoholic fatty liver disease
NFS: NAFLD fibrosis score
NOTCH2: Notch receptor 2
OVX: Ovariectomized
PCOS: Polycystic ovary syndrome
PCSK9: Proprotein convertase subtilisin kexin type 9
PEPCK: Phosphoenolpyruvate carboxykinase
PER2: Period circadian regulator 2
PPARα: Peroxisome proliferator activated receptor alpha
PNPLA3: Patatin like domain 3, 1-acylglycerol-3-phosphate O-acyltransferase
PPARGC1A: PPARG coactivator 1 alpha
PPM1L: Protein phosphatase, Mg^2+^/Mn^2+^ dependent 1L
PRKCH: Protein kinase C eta
PSORS1C1: Psoriasis susceptibility 1 candidate 1
PTEN: Phosphatase and tensin homolog
PXR: Pregnane X receptor
REV-ERBα: Nuclear receptor subfamily 1 group D member 1
RNA-seq: RNA-sequencing
RORγ: RAR-related orphan receptor gamma
ROS: Reactive oxygen species
SCN: Suprachiasmatic nucleus
SNP: Single nucleotide polymorphisms
SOCS3: Suppressor of cytokine signaling 3
SRC-2: Steroid receptor coactivator-2
SRY: Sex-determining region Y
STAT: Signal transducer and activator of transcription
SULT: Sulfotransferase
TCDD: 2,3,7,8-Tetrachlorodibenzo-p-dioxin
TCGA: The cancer genome atlas
TNFα: Tumor necrosis factor alpha
UGT: Glucuronosyltransferase
